# Neck Disability Index Is Better in Classification of Recovery after Whiplash Injury in Comparison with Ultrasound Shear Wave Elastography of Trapezius Muscle

**DOI:** 10.3390/diagnostics11112077

**Published:** 2021-11-10

**Authors:** Blaž Barun, Igor Barišić, Ana Krnić, Benjamin Benzon, Tonko Vlak, Jure Aljinović

**Affiliations:** 1 Institute of Physical and Rehabilitation Medicine with Rheumatology, University Hospital Split, Šoltanska 1, 21000 Split, Croatia; blaz.barun1@gmail.com (B.B.); tonkovlak@gmail.com (T.V.); 2 Clinical Department of Diagnostic and Interventional Radiology, University Hospital of Split, 21000 Split, Croatia; igorbarisic@net.hr (I.B.); a_krnic@yahoo.com (A.K.); 3 Departments of Anatomy, Histology and Embryology and Neuroscience, School of Medicine, University of Split, 21000 Split, Croatia; benzon.benjamin@gmail.com; 4 Department of Physical and Rehabilitation Medicine, School of Medicine, University of Split, 21000 Split, Croatia; 5 Department for Health Studies, University of Split, 21000 Split, Croatia

**Keywords:** car accident, stiffness, depression, neck pain, recovery

## Abstract

A prospective observational study comparing shear wave elastography (SWE) of trapezius muscle with Neck Disability Index (NDI) in a prediction model of health status six months after a whiplash injury. Both SWE values, measured by two radiologists, and NDI scores were obtained at baseline and after physical therapy (PT) (6-month period). Those values were compared with a 3-point Likert scale (no, partial or full recovery). Twenty-two subjects completed the study. A decrease in trapezius stiffness was detected by both radiologists, statistically significant for one (Δ10.1 kPa; *p* = 0.04) but not for the second radiologist (Δ8.63 kPa; *p* = 0.07). The measurements showed excellent intra-observer (ICC 0.75–0.94) and inter-observer (ICC 0.78–0.88) reliability. After six months, fully recovered patients showed lower NDI scores than partially recovered patients (Δ22.98; *p* < 0.001). SWE values did not differ with the recovery status (55.6 ± 9.7 vs. 57 ± 15.8, Δ1.45; *p* = 0.82). The single most accurate variable in classifying health status six months after whiplash injury was the relative change of NDI, and it showed the highest accuracy (73.9%) and low Akaike information criterion (AIC = 39.2). Overall, the most accurate classification was obtained when combining NDI and SWE after physical therapy with an accuracy of 77.3% and a decrease in AIC (32.8).

## 1. Introduction

Whiplash injury is the most common injury related to traffic accidents [1,2]. Approximately 30–50% of people experience chronic pain and disability following a whiplash injury, and 13–50% do not return to work six months after the injury; therefore, it becomes a great socioeconomic problem [2,3,4]. Although whiplash injury is common in modern society, and its incidence is increasing, the pathophysiological mechanism that leads to chronic disability and optimal treatment remains unclear [5]. Clinical guidelines identify the acute whiplash injury phase (90 days from the accident) as a critical period to classify patients according to the risk of developing chronic pain and disability. Patients at greater risk should be monitored more often, and their rehabilitation plans should be tailored individually [6,7].

Currently, subjective methods are used to assess the severity of the injury, prognosis, and recovery, with Neck Disability Index (NDI) being one of the most reliable and most commonly used ones [8,9,10,11].

There are no objective measurements used for the assessment of recovery following a whiplash injury of the neck. Data from previous studies showed that changes in cervical radiographs found after a car accident (straightening of cervical lordosis, kyphosis, or degenerative changes) are not suitable for estimating the severity of the injury and recovery prognosis [12,13]. The range of movement in the cervical spine, which can easily be measured, can vary up to 50% even on the same day, depending on the current pain level and patient’s compliance when performing tests [14].

Ultrasound shear wave elastography (SWE) is another research method of evaluating the severity of whiplash injury by measuring the muscle stiffness in the cervical region. It is used to analyze structural changes in different tissues, including muscles. This method allows both qualitative and quantitative evaluation of the elasticity characteristics of soft tissues and their alteration in traumatic lesions and degenerative pathology. Tissue stiffness is measured by the shear modulus and expressed in pressure units (kilopascals; kPa) [15].

It was previously published that SWE values of the trapezius muscle are increased in the first 90 days after a whiplash injury compared to the healthy control group (75 vs. 50 kPa), with a sensitivity of 90% and specificity of 72%. That study was conducted on 75 participants in each group, with the intra-observer reliability of SWE values of stiffness of the trapezius muscle being 0.8. The authors suggested that more participants were needed to have more precise results, and inter-observer reliability was not calculated [16]. We were unable to find published reports on how SWE values change after physical therapy (PT) and studies investigating SWE as a potential prognostic factor of recovery after a whiplash injury.

The objective of this study was to analyze the feasibility of the usage of baseline or follow-up SWE values or their change in the classification of recovery six months after the whiplash injury and compare it with NDI.

## 2. Methods

### 2.1. Study Design

This prospective observational study was conducted from January 2020 to February 2021 at the Institute of Physical Medicine and Rehabilitation, University Hospital Split in Croatia and was approved by the Ethical Committee of the University Hospital Split. All participants were informed about the study design, and informed consent was obtained in written form.

### 2.2. Participants

Participants in this study were all symptomatic whiplash injury patients referred to a physiatrist that complied with inclusion and exclusion criteria and were willing to participate in the study. Enrolled subjects met the following inclusion criteria: patients older than 18 who sustained whiplash injury of the neck in a car accident as drivers or co-drivers, examined by physical medicine and rehabilitation (PMR) specialist within 90 days from the accident, and diagnosed with whiplash injury of the neck. Participants were excluded if they sustained bone fractures or spinal cord injury in an accident, had an accident in any vehicle other than a car, were treated with malignant disease in the last 5 years, or were treated with a severe mental illness (psychosis or major depressive disorder).

### 2.3. Variables

Patients following whiplash injury undertook the standardized physical therapy program individually at the outpatient clinic guided by a physiotherapist. It included supervised exercise, transcutaneous electrical nerve stimulation (TENS), therapeutic ultrasound, advice on posture, pain relief methods, and early return to daily activities.

The physical therapy program consisted of two parts: a 2-week program with therapies 5 times a week, followed by a 3-week break, and then another 2 weeks of therapies.

TENS and ultrasound were applied before starting the exercise. TENS (100 Hz) was administered for 20 min on the paravertebral musculature of the neck. Pulsed ultrasound (0.8 W/cm^2^ with 1 MHz ultrasound probe) was applied for 5 min on trapezius muscles.

Initially, the exercise program focused on breathing exercises, gentle range of motion exercises, deep neck flexor activation, and stretching exercises. The importance of good posture was emphasized to facilitate deep cervical muscle function. Toward the end of the 2-week exercise period, participants were encouraged to continue exercise at home. The physician gave them written and illustrated material explaining the home exercise program. After three weeks, they came back for another 2-week program. Exercise then progressed with low isometric resistance, endurance training of shoulders, and an increase in the number of repetitions. Progressions were adjusted to each patient according to their symptoms and capability. After completing the entire program, participants were encouraged to continue exercising at home, avoid the aggravation of pain, and avoid any other physical treatments for their neck disorder during the six months of participating in this study.

### 2.4. Outcomes

The primary outcome measure of this study was the stiffness of the trapezius muscle obtained with SWE.

Secondary outcome measures included physical functioning assessed with NDI and depression level before the PT assessed with Patient Health Questionnaire 9 (PHQ-9). A three-point Likert scale with the values of no, partial, or full recovery was used for health assessment as a patient-perceived recovery scale (PPR).

### 2.5. Data Measurement

Two radiologists blinded to the status of the patient and blinded from each other performed ultrasound (US) SWE of the trapezius muscle. We used a multi-frequency linear probe (2–10 MHz, Aixplorer Multiwave™ System, Supersonic Imagine, Aix-en-Provence, France), and the level of muscle stiffness was measured by shear modulus and expressed in pressure units (kPa) with absolute elasticity values ranging from 0–300 kPa.

The patient sat on a chair with the relaxed shoulder girdle and arms in supination, resting on thighs. The radiology specialist put the US probe longitudinally to the muscle belly of the upper portion of the trapezius muscle in the shoulder region using minimal pressure of the US probe against the skin, identified the size of the circular regions of interest (ROIs) as the thickness of the upper trapezius, and measured mean stiffness. Measurement was repeated 3 times for each muscle. Separately, within 30 min, another radiology specialist performed the same procedure. The difference between left and right trapezius muscle was calculated for both radiologists with no significant difference. The mean of all 6 measurements (left and right trapezius) was calculated. The difference between measures obtained at baseline and 6-month follow-up was determined for each radiology specialist separately.

NDI-CRO, previously validated in Croatian, was used to quantify disability [17]. The total NDI score was divided by the maximal possible score and expressed in percentages (NDI%). Values from 0–8% are regarded as no disability, 10–28% mild disability, 30–48% moderate disability, 50–68% severe disability, and 70–100% total disability.

The level of depression was determined by the Patient Health Questionnaire 9 (PHQ-9) index: scores of 5–10, 11–15, 16–20, and >21 representing mild, moderate, moderately severe, and severe depression, respectively.

### 2.6. Study Size

The sample size was calculated based on the pilot study (n = 14 pairs). Given the observed differences in the pilot study, we calculated the needed sample size (n = 20 pairs) to obtain the power of 80% at the α = 0.05. The sample size was calculated based on paired *t*-test in G*Power Software Version 3.1.9.6 [18]. The final number of participants was calculated as 50% more than the power analyses required (n = 30).

### 2.7. Statistical Methods

Continuous data are presented as average and standard deviation. Intra-observer reliability was assessed by interclass correlation (ICC) with model 3.1 and inter-observer reliability with model 2.1 of Shrout and Fleiss and interpreted according to Cicchetti [19]. The differences in continuous variables were assessed by *t*-test for paired samples. Statistical measures of evidence are presented as effect size and its 95% confidence interval (CI) or its standard deviation (SD), *p*-values, R^2^, and corrected Akaike information criteria (AIC). For building of the classification model, we used ordinal logistic regression in Gretl software [20]. Model accuracy is defined as ratio of correct predictions and all predictions made by model. All other statistical analyses were done in Past3 Software (Hammer, O. et al., 2001. PAST).

## 3. Results

**Figure 1:**
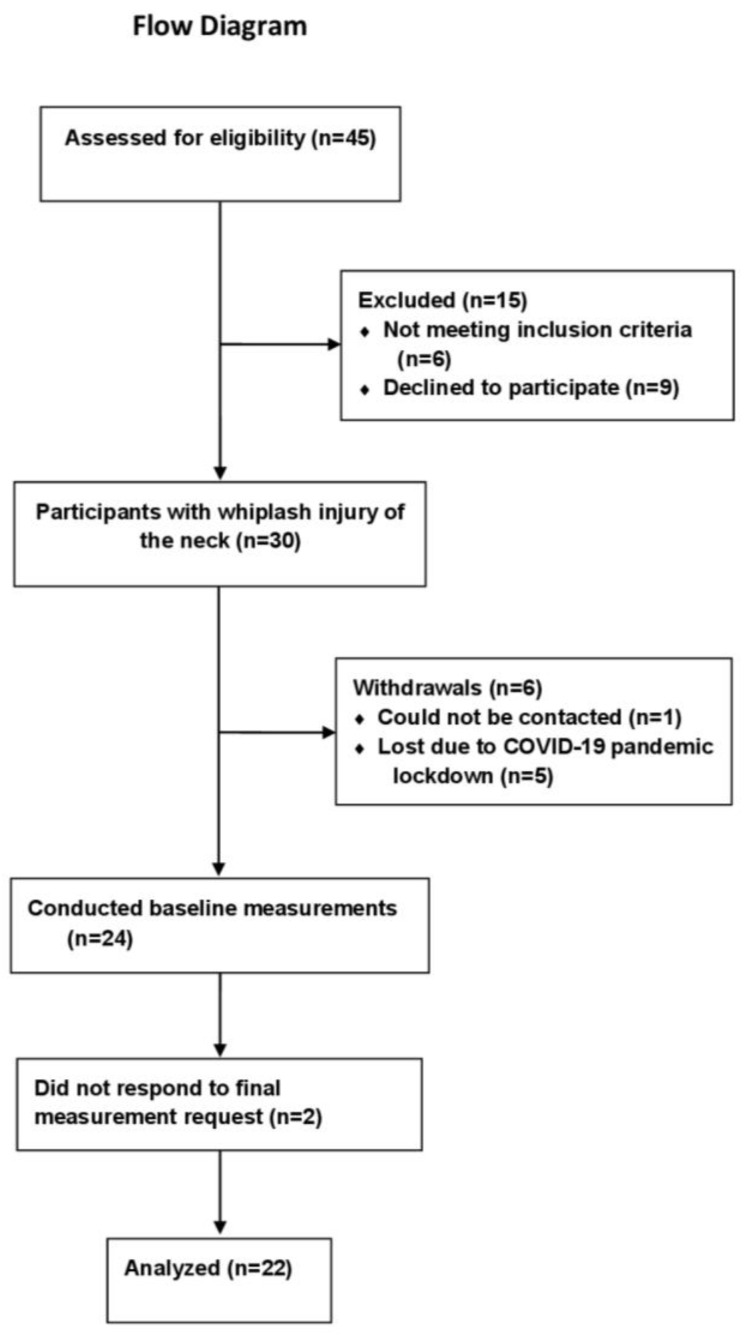
Flow of participants through the study.

A flowchart of the 45 whiplash injury patients that were assessed for eligibility is shown in Figure 1. The baseline characteristics of the participants are shown in Table 1.

A complete follow-up examination six months after whiplash injury was obtained in 22 participants (12 women and 10 men; from 23 to 58 years of age; the mean age was 38.6 ± 11 years). Another two participants did not obtain SWE after six months, and three did not fill out NDI at six-month follow-up. All patients were drivers or co-drivers and used a seatbelt in a car accident. All subjects obtained radiographs of the cervical spine; thirteen were reported as normal, fourteen as cervical spine straightening, and three as a segmental kyphosis.

The difference in muscle stiffness between left and right trapezius before physical therapy and the difference in muscle stiffness between left and right trapezius after physical therapy was calculated for both radiologists. There was no statistical difference between left and right trapezius muscle stiffness for the first examiner at baseline (Δ = 4.84 kPa; *p* = 0.34) and six-month follow-up (Δ = 1.2 kPa; *p* = 0.72) nor for the second examiner at baseline (Δ = 7.84 kPa; *p* = 0.053) and six-month follow-up (Δ = 0.4 kPa; *p* = 0.89). Since there was no difference between the left and the right trapezius muscle’s stiffness in the same person at the analyzed time points, for further statistical analyses, the mean of six measurements was used (three measurements of the right trapezius and three measurements of the left trapezius).

**Figure 2:**
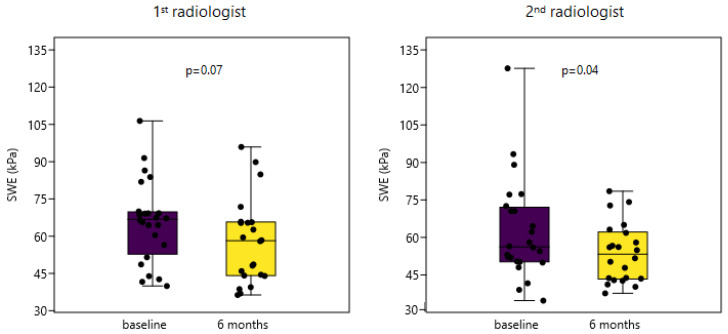
The difference of shear wave elastography (SWE) at baseline and 6-month follow-up measured by two radiologists. kPa, kilopascals.

The difference in trapezius muscle stiffness measured at baseline and six-month follow-up was analyzed. Unlike measurements obtained by the first radiologist, which did not reach statistical significance (Δ = 8.63 kPa; *p* = 0.07), measurement differences obtained by the second radiologist were statistically significant (Δ = 10.1 kPa; *p* = 0.04)(Figure 2).

Furthermore, post-hoc power analysis was calculated on n = 22 (pairs), with an α = 0.05 and power of 0.8. Given the observed differences between timepoints, the second examiner needed 21 pairs of measurements to reach statistical significance, while the first examiner was underpowered (24 pairs needed). However, if the SWE differences between timepoints for each patient are compared between radiologists, then practically no differences can be found (Δ = 1.47 kPa; *p* = 0.65).

Intra-observer reliability at baseline and at six-month follow-up showed ICC between 0.75 and 0.94, which indicates excellent reliability for both radiologists and all muscles (Table 2).

Inter-observer reliability of SWE measurements of left and right trapezius muscle at baseline and six-month follow-up indicates excellent reliability by inter-class correlation (ICC varied from 0.78 to 0.88) (Table 3). Given the excellent inter-observer reliability and the finding that there are no practical differences between radiologists in measuring the individual patient difference in SWE between baseline and six-month follow-up, we decided to include in further analyses SWE values that are arithmetic average of values measured by two radiologists.

**Figure 3:**
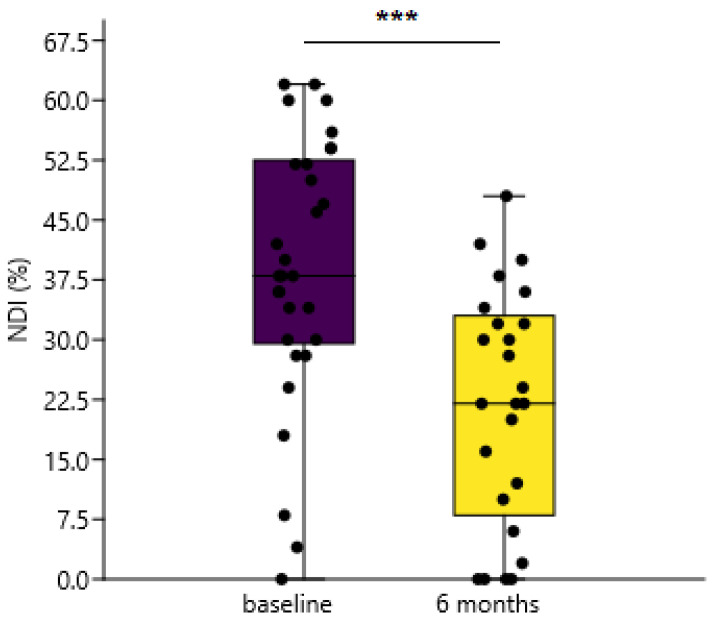
Statistically significant differences in NDI measured at baseline and 6-month follow-up, *** *p* < 0.0001, *t*-test (mean ± 95% CI), NDI showed in percentages of the maximum score.

NDI at baseline and at six-month follow-up was calculated. Mean NDI score (in percentages) was significantly lower at six-month follow-up in contrast to the baseline measurement when analyzing all the patients together (Δ = 15.28%, CI (8.6332%, 21.927%), *p* < 0.0001), (Figure 3).

Using PPR, total recovery was reported by 42% (n = 10) of patients, while 46% (n = 11) said that they were partially recovered and 12% (n = 3) that they were not recovered.

**Figure 4:**
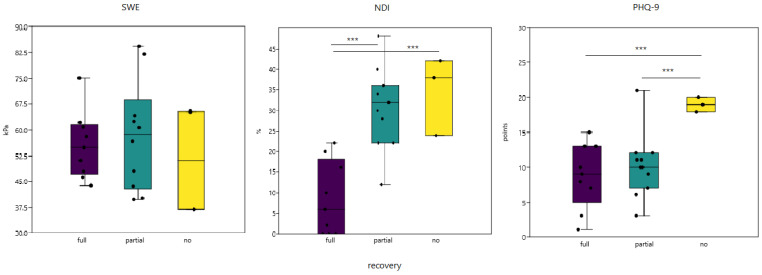
Differences in endpoint shear wave elastography measured as an average of both radiologists, Neck Disability index after 6 months, and initial PHQ-9 in comparison to patient perceived recovery status, *** *p* < 0.0001, *t*-test (mean ± 95% CI).

Upon further subdivision of patients regarding PPR, NDI scores at six-month follow-up differed between groups: fully recovered (mean 8.4 ± 8.9), partially recovered (mean 30.5 ± 9.67), and not recovered group (mean 34.7 ± 9.45). The correlation between the NDI score obtained at six-month follow-up, and perceived recovery type formed a significant quadratic trend (R^2^ = 62.5%; *p* < 0.001). Furthermore, when no recovery and partial recovery groups were merged, together, they significantly differed from the fully recovered group (Δ = 22.98; *p* < 0.001) (Figure 4).

SWE values obtained at six-month follow-up stratified by PPR groups were calculated for both radiologists and as the average of latter (Figure 4). Measured by the first examiner, SWE values did not differ in relation to recovery status (49.1 ± 8.3 vs. 57.6 ± 13.09, Δ = 8.5; *p* = 0.08). In contrast, fully recovered patients had lower SWE values compared to those who recovered partially and did not recover at all when measured by the second radiologist (48.1 ± 10.7 vs. 65.7 ± 17.64, Δ = 17.55; *p* = 0.01). On the other hand, when the same groups were compared in terms of pooled SWE from both radiologists, no difference could be found (Δ = 1.45, *p* = 0.81).

Initial PHQ-9 scores were compared between PPR groups, and significantly higher PHQ-9 scores were found in participants who answered that they did not recover than those who reported complete or partial recovery (mean 9.3 ± 4.69 and 10.2 ± 4.69 vs. 19 ± 1; *p* < 0.001) (Figure 4).

Models classifying patient-perceived recovery were calculated (Table 4). Between all measures (NDI, PHQ-9, and SWE) obtained before and after physical therapy, the relative change of NDI $\frac{{\mathrm{NDI}}_{\mathrm{after}\;\mathrm{PT}}\;-\;{\mathrm{NDI}}_{\mathrm{baseline}}}{{\mathrm{NDI}}_{\mathrm{baseline}}}\cdot 100$ showed to be the most accurate classifier of recovery (accuracy 73.9%, AIC 39.21), followed by NDI measured after the therapy (accuracy 69.6%, AIC 33.74). SWE before and after physical therapy and its change showed accuracy from 40–64% but with higher AIC values (≈46), which limits their usage (Table 4). When a combination of potential classifiers was calculated, SWE obtained after the therapy combined with NDI after physical therapy raised accuracy to 77.3% with AIC 32.85 (Table 4).

Model coefficients for best predictive models (relative ΔNDI and combined SWE after PT with NDI after PT) with cut-off values for allocating into recovery groups are shown in Table 5.

## 4. Discussion

Our study was the first to compare SWE values at baseline and after the follow-up period of six months. We also assessed the value of SWE as a prognostic or classification tool in the recovery of whiplash injury patients. In this study, lower SWE values were measured in patients after performing PT at a six-month follow-up. Excellent intra- and inter-observer reliability of SWE in measuring the trapezius muscle stiffness at baseline and six-month follow-up was found. These findings are consistent with those from the studies assessing intra- and inter-observer reliability of SWE of the upper trapezius muscle and other muscles both in healthy individuals and in pathological conditions, such as cerebral palsy [21,22,23,24,25,26].

We did not find a difference in stiffness between left and right trapezius muscles in whiplash injury patients before and after PT. This is in accordance with the results from our previous study [16] but differs from Zhang et al. [27], which reported a significant difference between dominant and non-dominant upper trapezius muscle. This difference could be due to different study populations. Zhang et al. obtained measurements on 20 healthy young male participants [27]. We assume that factors such as injury, age, gender, and work position could affect these results.

NDI has already been described in the literature as a good prognostic factor of recovery after the injury [28,29,30,31]. In this study, NDI has proven to be a better outcome classifier, with higher accuracy and lower possibility of error than SWE. The best health status classification can be made from the relative change of NDI (almost 74%) with an AIC value of 39.2.

However, six-month follow-up SWE values combined with NDI raised the accuracy of classifying outcomes with a further decrease in error risk (77.3%, AIC 34.21). Consequently, SWE after PT could be used with NDI after PT for estimating recovery after a whiplash injury, keeping in mind that SWE requires the work of an experienced operator with adequate equipment, which is expensive and not always available [32].

When analyzed individually, initial SWE values of upper trapezius muscle showed a high risk of error in predicting outcome and are unsuitable for being used as a prognostic tool. Due to the low accuracy and a high risk of error when classifying the patients in different recovery groups, SWE values obtained after six months and the change of SWE values showed to be inadequate for recovery status classification.

Postural changes in patients with neck and shoulder complaints lead to greater stiffness of the upper trapezius muscle [33,34]. Various therapies can lower trapezius stiffness in the short term [35,36]. However, it is important to emphasize that muscle stiffness in healthy subjects does not vary in time, even after a long-term exercise program [37,38,39]. Although trapezius stiffness in whiplash injury patients decreased after PT at a six-month follow-up, it is difficult to determine the clinical implications of the observed changes in SWE values. We assume that the change in the trapezius stiffness addresses just one part of the recovery. Hence, it is not as sensitive as NDI, which engages more components involved in the recovery. This is supported by literature that affirms other factors influencing the outcome after the injury, such as psychological ones [40,41].

## 5. Limitations

This study requires consideration of a few limitations. First, a major limitation was a high dropout rate of 27%. The study was conducted during the unexpected COVID-19 lockdown, and patients were advised to attend only urgent medical examinations. Although we enrolled 50% more participants than proposed after performing power analysis, lowering of the trapezius stiffness did not reach statistical significance for the first radiologist. Post-hoc power analysis showed that three more participants were needed. Higher values of muscle stiffness can be measured if an individual intentionally contracts the trapezius muscle, so to minimize this error, all participants were instructed to relax, and SWE was performed after confirmation of no muscle contraction on the B-mode image. Additionally, the question of patients’ home exercise adherence should be included in future studies and tested with patient recovery status.

## 6. Conclusions

We have found that SWE is not as good a classifier of recovery as NDI six months after a whiplash injury. Furthermore, NDI showed both better accuracy and a smaller margin of error. The most accurate variable in the classification of recovery was the relative change of NDI, and the overall best classification was obtained when combining NDI and SWE after physical therapy.

## Figures and Tables

**Table 1:** Demographic and baseline characteristics of whiplash injury patients that met eligibility criteria.

Patients (n = 30)	
Age, Median (IQR)	37.5 (31–52)
Female, n (%)	17 (57)
Time from accident, Mean ± SD	30.3 ± 12.8
Drivers, n (%)	26 (87)
Doctor’s office visits, Mean ± SD	3.9 ± 1.2
Absence from work, n (%)	20 (67)
VAS of pain, Mean ± SD	5.1 ± 2.7
Analgesic drugs use, n (%)	
Occasionally	5 (17)
1 time/day	24 (80)
2 or more times/day	1 (3)
Myorelaxant drugs use, n (%)	18 (60)

IQR, interquartile range.

**Table 2:** Intra-observer reliability of elastography measurements in trapezius muscles before and after physical therapy.

Elastography of Muscle	Radiologist No1		Radiologist No2	
	ICC Coefficient	95% CI	ICC Coefficient	95% CI
Before PT				
Trapezius R	0.79	(0.636, 0.8946)	0.75	(0.6779, 0.9091)
Trapezius L	0.75	(0.5839, 0.8755)	0.85	(0.7388, 0.9289)
After PT				
Trapezius R	0.94	(0.9001, 0.9768)	0.93	[0.8716, 0.9698)
Trapezius L	0.88	(0.782, 0.9459)	0.88	[0.7888, 0.9478)

Legend: R, right; L, left; ICC, inter-class correlation; PT, physical therapy.

**Table 3:** Inter-observer reliability of elastography measurements in trapezius muscles before and after physical therapy.

Elastography of Muscle	ICC Coefficient	95% CI
Before PT		
Trapezius R	0.8787	(0.7211, 0.9474)
Trapezius L	0.7828	(0.4955, 0.9062)
After PT		
Trapezius R	0.795	(0.5131, 0.9144)
Trapezius L	0.7839	(0.4857, 0.9099)

Legend: R, right; L, left; ICC, inter-class correlation; PT, physical therapy.

**Table 4:** Models predicting patient-perceived recovery.

Variables in Model	AIC	Accuracy
SWE before PT	46.6	63.6%
SWE after PT	46.6	40.9%
ΔSWE	46.47	50%
Relative ΔSWE	46.62	50%
NDI (%) before PT	49.66	56.5%
NDI (%) after PT	33.74	69.6%
ΔNDI	47.37	60.9%
Relative ΔNDI	39.21	73.9%
PHQ9	44.54	47.8%
PHQ9 and SWE after PT	44.29	54.5%
NDI (%) after PT and PHQ9	34.02	69.6%
NDI (%) after PT and SWE after PT	32.85	77.3%
SWE after PT, NDI (%) after PT and PHQ9	34.2	72.7%

Legend: AIC, Akaike information criterion (AIC) is an estimator of prediction error; SWE, shear wave elastography; PT, physical therapy; NDI, Neck Disability Index; PHQ-9, Patient Health Questionnaire 9; ΔSWE, change from 6-month vs. baseline measurement; ΔNDI, change from 6-month vs. baseline measurement; Relative ΔNDI (%) = $\frac{{\mathrm{NDI}}_{\text{after}\;\mathrm{PT}}\;-\;{\mathrm{NDI}}_{\text{baseline}}}{{\mathrm{NDI}}_{\text{baseline}}}\cdot 100$; Relative ΔSWE (%) = $\frac{{\mathrm{SWE}}_{\text{after}\;\mathrm{PT}}\;-\;{\mathrm{SWE}}_{\text{baseline}}}{{\mathrm{SWE}}_{\text{baseline}}}\cdot 100$; analysis was done on pooled (averaged) SWE data from both radiologists.

**Table 5:** Model coefficients for most accurate models.

Model with Single Variable		
Variable (*x*)	*Coefficient (β)*	*Std. Error*
Relative ΔNDI	0.048	0.017
cut1	−2.72	1.08
cut2	1.59	0.83
**Model with Combined Variables**		
Variable *(x*)	*Coefficient (β)*	*Std. Error*
SWE after PT	−0.025	0.031
NDI (%) after PT	0.13	0.07
cut1	0.15	2.64
cut2	4.8	2.79

Legend: dependent variable (*y*) value less then cut1 implies full recovery, between cut1 and cut2 partial recovery, and above cut2 no recovery. Model equation is ordinal logistic regression equation ${y=\beta }_{1}x_{1}{+\beta }_{2}x_{2}$. Thus, for model with single variable, the equation is *y* = 0.048 · relative ΔNDI, where relative ΔNDI = $\frac{{\mathrm{NDI}}_{\text{after}\;\mathrm{PT}}\;-\;{\mathrm{NDI}}_{\text{before}\;\mathrm{PT}}}{{\mathrm{NDI}}_{\text{after}\;\mathrm{PT}}}\cdot 100$; for model with combined variables, the equation is $y\;=\;-0.025\cdot {\mathrm{SWE}}_{\mathrm{after}\;\mathrm{PT}}\;+\;0.013\cdot {\mathrm{NDI}\;(\%)}_{\text{after}\;\mathrm{PT}}$.

## Data Availability

Data are available upon request.
